# In celebration of Hispanic women in neuroscience

**DOI:** 10.3389/fnana.2023.1179254

**Published:** 2023-05-09

**Authors:** María Jimena Salcedo-Arellano, Adriana P. Pantoja, Estela M. Muñoz, Verónica Martínez-Cerdeño

**Affiliations:** ^1^Department of Pathology and Laboratory Medicine, University of California Davis School of Medicine, Sacramento, CA, United States; ^2^Medical Investigation of Neurodevelopmental Disorders (MIND) Institute, University of California, Davis, Sacramento, CA, United States; ^3^Instituto de Histología y Embriología de Mendoza “Dr. Mario H. Burgos” (IHEM), Universidad Nacional de Cuyo (UNCuyo), Consejo Nacional de Investigaciones Científicas y Técnicas (CONICET), Mendoza, Argentina

**Keywords:** neuroscience, diversity, women in neuroscience, neuroscience research, Hispanic women

## 1. Introduction

While historically the role of women in science was practically non-existent or ignored, during the late 20th and early 21st centuries the participation of women in science increased. Data for women are encouraging. UNESCO indicates that 29% of all researchers in the world are women. The figure is more reassuring among Hispanic countries, where the average percentage of women in science reaches 45%. Neuroscience is a notable field where the representation of women is reduced compared to men, and of these women very few occupy positions of higher responsibility. A study by the International Brain Research Organization (IBRO) Latin America Regional Committee (LARC) indicated that women face numerous challenges in terms of equality at different stages of education and career paths. They collected data from 750 Latin American neuroscientists associated with IBRO-LARC, and investigated the development of their scientific careers in parallel with family life, their perceptions of obstacles to success, and their ideas to overcome inequalities. They concluded the existence of horizontal (scientific area) and vertical (training and research positions) segregation, a glass ceiling (high-rank positions), discrimination, and other serious obstacles. The Spanish Society for Neuroscience (Sociedad Española de Neurociencias) warns us about the current belief that women and men are equally treated in Neuroscience, and a type of discrimination that is hidden and difficult to perceive; while in Spain the number of women graduates is steadily increasing, the number of women occupying higher up positions has been unchanged over the last 30 years.

**FIGURE 1:**
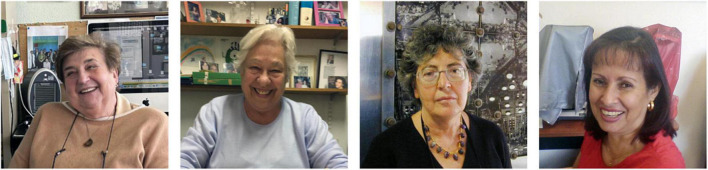
From left to right: Isabel de Andrés Ph.D., Giannina Pasquini, Ph.D., Herminia Pasantes, Ph. D. and Martha Isabel Escobar, M.Sc.

Herein we invite you learn and reflect upon the success stories of four remarkable Hispanic women neuroscientists who have opened doors for many of us reading this manuscript, Isabel de Andrés, Giannina Pasquini, Herminia Pasantes, and Martha Isabel Escobar (Figure 1). During our interview, we wanted to emphasize their greatest accomplishments, their unique pathway toward becoming neuroscientists, and demonstrate how they have left their mark on the field of Neuroscience.

## 2. Discussion

### 2.1. Isabel de Andrés

Isabel de Andrés, Ph.D. is an inspiring Spanish neuroscientist and Emeritus Professor at the Universidad Autónoma de Madrid (UAM). She was born into a family that had gone through the vicissitudes of the Spanish civil war, with no tradition of university studies. Those were difficult years, but her parents thought that the best legacy they could give to their children was to provide them with the opportunity to pursue higher education. With a lot of sacrifice, her parents sent Isabel and her older brother off to a university. While being very aware of her brother’s progress, her parents did not show the same interest to follow on her achievements. Little did they know she would go on to become one of the greatest Spanish scientists of all time.

Dr. de Andrés graduated with a degree in Biology in 1964 and obtained her Ph.D. in 1975, both at the Complutense University in Madrid. In 1971 she joined Professor Reinoso-Suárez who was interested in neural connection tracing studies. Under Dr. Reinoso-Suárez mentorship she began studying Nissl and Fink-Heimer cat brain histological sections to detect degenerated axons after having produced lesions in the nervous tissue. Dr. Reinoso-Suárez also studied the mechanisms of sleep and wakefulness, and it was during this period that she became interested in the anatomy of sleep and began her Ph.D. on this topic a year later, when the first polygraph arrived to UAM. She then spent 2 years in the University of California, Los Angeles where she studied the effect of opioids on sleep. Since then, she has worked on sleep physiology and anatomy of brain regions that support sleep. In 1987 she became the first Biology graduate woman to become full Professor in a Medical School in Spain.

Dr. de Andrés current research focuses on the rapid-eye-movement (REM) sleep mechanisms mediated by the pontine tegmentum, and in the prosencephalic and brainstem basis of opiates actions on sleep. During our interview she told us, “I am proud of all the work that has been carried out in my sleep laboratory, but I think I am the proudest of the results obtained in collaboration with my colleagues that allowed us to propose that the ventral region of the pontine tegmentum is a trigger region for REM sleep.” Other important contributions are the finding of a distinct participation of the forebrain and the brainstem in REM sleep homeostasis and in mechanisms of action of opioids in sleep and electroencephalogram.

Dr. de Andrés has not only witnessed but also participated in historical Neuroscience moments in Spain, including the beginnings of the UAM and of the Spanish Society of Neuroscience (SENC). She recalls how the field of Neuroscience was first introduced by the newborn UAM by integrating the fields of Neuroanatomy and Neurophysiology, thanks to professors Dr. Reinoso-Suárez from Spain and Dr. Garcia-Austt from Ecuador. These two researchers decided to create the SENC, where Isabel worked as secretary. It took 6 years to finally establish the society in 1985. Dr. de Andrés oversaw registering the first society laws and created the first directive group. To date, the SENC is one of the scientific societies with more members in Spain.

Her work goes beyond her science. She has been an excellent teacher and mentor. Her goal has been to ensure that her students receive the best education possible. Throughout her career, she mentored many doctoral students. She was the director of the doctorate program of Neuroscience at the UAM for almost 15 years, and the director of the master program of Neuroscience until she retired. She is pleased with the prestige that the UAM graduate program has among the young neuroscientists in the country. Her passion and dedication for research and teaching make her an ideal role model to future generations of young women scientists to follow.

### 2.2. Juana María “Giannina” Pasquini

Dr. Pasquini, is one of the most inspiring female neuroscientists of Argentina. She is an Emeritus Professor of the University of Buenos Aires (UBA), and Ad-honorem Superior Investigator of the National Scientific and Technical Research Council. Dr. Pasquini received these high honors for her passion and tireless dedication during her life’s work in science, education, and management. Her achievements are even more impressive when considering the tumultuous political and social changes Argentina endured, often leading to ups and downs in the country’s educational and scientific policies.

Giannina graduated in Pharmacy and Biochemistry at UBA. In 1964, she began her long multi-step career that intertwined both teaching and scientific research. The latter under the mentorship of Professor Dr. Carlos J. Gómez. After completing her doctorate, Dr. Pasquini went on to develop her own very prolific mentoring career, which includes more than twenty mentees. Several of them have successfully moved on to establish notable careers of their own.

To this day, her enthusiasm and dedication for mentoring undergraduate and postgraduate students continues. In addition, Giannina has developed many productive collaborations, both nationally and internationally. She points to her loving husband, Dr. Eduardo Soto, among her collaborators. She considers her work with mentees and colleagues as her most valuable and long-lasting accomplishment. They, in turn, recognize her not only as an inspiring scientist but also as an affable woman who is always ready to engage in an interesting conversation.

Dr. Pasquini’s contributions to Neurochemistry include remarkable studies in collaboration with Dr. Soto and Dr. De Robertis about the cholinergic receptor at synaptic structures, also the effects of thyroid hormones during brain development, myelin chemistry and synthesis, the role of iron and its deficiency in oligodendrocyte differentiation and myelinogenesis, and more recently, the participation of the immune system in myelin turnover, in collaboration with Drs. Rabinovich and L. Pasquini. In addition, she is applying her expertise to study Multiple Sclerosis with Dr. Correale.

Another milestone that deserves mentioning is Giannina’s work as an administrator. She was the first democratically elected Dean of the Faculty of Pharmacy and Biochemistry in 1986, and the first woman who had the role of Dean in the entire UBA. She produced enormous positive changes toward a modern, scientific, and democratic University. She also has worked hard to promote Neurochemistry and Neuroscience in the region and in the rest of the world, through active participation in several initiatives, such as the Argentine Society for Neurochemistry (currently Argentine Society for Research in Neuroscience), IBRO-LARC, and the Glia Club, among others. Giannina was selected to present the first plenary Marthe Vogt Lecture at the 2019 International Society for Neurochemistry meeting in recognition of her outstanding career.

Dr. Giannina Pasquini came from humble beginnings as a young girl of the small farming town of Huinca Renancó, in the heart of Argentina. Coming from a small town, she fearlessly conquered the big city of Buenos Aires, and its prestigious UBA. Undoubtedly, she has overcome many obstacles along the way to become the leader for the field of Neuroscience that she is today. For these reasons, she knows better than many of us that much more needs to be done so that more women in science are recognized for their achievements and are promoted into positions of leadership. Dr. Pasquini’s story and others like it have and will continue to inspire the right people to make the right changes to achieve these goals.

### 2.3. Herminia Pasantes

Dr. Herminia Pasantes is a highly honored Mexican neuroscientist. She is currently an Emeritus Researcher at the Universidad Nacional Autónoma de México (UNAM) and a writer. She serves as an inspiration and model to women in Neuroscience around the world. Her path to science was driven by her own curiosity and her love of literature. During her generation, a woman’s success was based on marriage and starting a family. However, for Herminia, since she wore thick, “unattractive” glasses at a young age, her parents told her she wouldn’t get married and instead to focus on her academics. At that moment, her fate toward academia was sealed.

She discovered her passion for science at a very young age. Being the first person in her family to go to college, she graduated from UNAM with a degree in Biology where she did research on embryology. Soon after she received her master’s in Biochemistry at UNAM. Here she worked under the mentorship of Dr. Guillermo Massieu, who would carry out pioneering research in Neurochemistry. Later, she received her doctorate from the Center of Neurochemistry at the University of Strasbourg in France under the supervision of Dr. Paul Mandel. She describes herself as hard working, dedicated, and competitive in the laboratory. She attributes part of her success to her mentors who taught her how to balance between intuition and reasoning for experimental design and the rigor in the analysis of the results. Dr. Pasantes explained that one of the most rewarding parts of her career is being able to mentor and create connections with her students. She believes that great mentorship is imperative for a person’s success. Under her supervision, nineteen students received their doctorates. Most of these mentees are now independent researchers in Mexico and abroad.

Dr. Pasantes is well known for her discovery of taurine as an osmolyte in volume regulation in the retina and in brain cells. Her work helped clarify the purpose of why there are high taurine levels in all cells and their purpose in neurons and astrocytes, despite their metabolic inertness. She helped contribute to the knowledge of the mechanisms of taurine mobilization in response to osmotic changes in the brain. Her laboratory focused on the mechanisms that regulate the volume of taurine in the cells of the brain and the physiopathology of neurons and glial cells with an emphasis on the control of cell volume. They have also delved in the neuroprotective actions of taurine, although the mechanism of action is still not well known.

Herminia was the first woman who was awarded Mexico’s “National Science and Arts Award” in the realm of physical, mathematical, and natural sciences. This award is the highest recognition granted by the government of the republic of Mexico to citizens whose career has contributed to science, art, and technology. She is a member of the Sistema Nacional de Investigadores III (National System of Researchers), a government agency in Mexico that promotes the quantity and quality of research. Level III recognizes researchers who have made momentous contributions to their respective fields.

Dr. Pasantes has paved the way for many women neuroscientists and her story alone is an inspiration to women. She expressed she never felt incapable in the laboratory; however, she experienced a lot of misogyny. Women were considered at the time a “bad investment.” She had to prove that she was just as capable, if not more, than a man. The biggest obstacle she faced was being denied the opportunity to apply to a doctorate program because of the birth of her daughter. However, this led her to completing her Ph.D. at Strasbourg which led her to having a very successful career and her greatest contributions to science.

During our interview we asked her: “what would be your advice to women who are thinking or wanting to go into the field of research?” She responded, “Look at me, use me as an example. It is possible to do it all. The time I spent with my children was of absolute dedication. I always prepared homemade porridge for them, I always told them a story before bed, and I did very well in the laboratory. Entonces ¡sí se puede!”

### 2.4. Martha Isabel Escobar

Martha Isabel Escobar, M.Sc. is an outstanding Colombian neuroscientist. She completed a degree in Biology from the Universidad del Valle (UV), Colombia in 1974, and a M.Sc. in Human Morphology in 1978. At the time, doctoral programs had not been established in Colombia, the first to be approved in the country in 1986 were in the fields of Mathematics and Physics. Shortly after obtaining her graduate degree, Martha started a career as professor and researcher at the UV, institution she served until her retirement. “When you have the possibility, as in my case, of studying Biomedical Sciences, the doors to the study of the human body are open to you. In my case, I could either be a histologist, an anatomist, an embryologist, or a neuroanatomist. And in all that sea of possibilities, the option of being able to explore even a little into the knowledge of the human brain was a dream. A dream that came true and for which I feel very grateful. I was able to meet researchers, professors and students from different national and international universities, with which we formed a team with no other interest than to continue advancing in Neuroscience.”

As a bright early career scientist, Professor Escobar was invited through a program funded by the Inter-American Development Bank to complete a fellowship in Neuroscience at Harvard University, Eunice Kennedy Shriver Center under the mentorship of Dr. Verne Caviness, where she shared her work with her husband, Professor Hernán Pimienta and Dr. Kenneth Kosik, currently a Harriman Professor at the University of California, Santa Barbara. Upon her return to Colombia, she joined the Department of Morphology as Professor of Neuroanatomy and devoted her career to educate generations of undergraduate and postgraduate students. She was a pioneer in the field of brain research in Colombia and contributed to the establishment of UV’s first doctoral program in Biomedical Sciences with an emphasis in Neuroscience in 1997, and the Center for Brain Studies (CBS). She served as Vice Dean for Research and as Director of the CBS. She received recognition for academic excellence by the Foundation for Higher Education, and became a Distinguished Professor in 2009.

During her career in Neuroscience, Professor Escobar worked alongside her beloved late husband. Their line of research began with the study of the cytoarchitecture of the cerebral cortex, specifically the organization of the apical dendrites of pyramidal cells. “When we started our research, the use of monoclonal antibodies with immunohistochemical methods was emerging.” In collaboration with Drs. Caviness and Kosik, they defined a model of normal dendritic organization using a monoclonal antibody against microtubule-associated protein 2 as selective and sensitive marker for neocortical dendrites, which is widely used in studies investigating the cerebral cortex. Their research group continues to carry out descriptive evaluations of brain tissue obtained from patients who suffered traumatic brain injury focusing on indicators of cell death and damage in response to acute and subacute insults. These studies have also identified microscopic variants of the expression of the lesion not observed in neuroimaging or during decompressive surgery. In addition, they confirmed the link between immunoexpression of some biomarkers with cell loss and characterized the differential response of subpopulations of cortical GABAergic interneurons. The latter has been very important in the study of post-traumatic epilepsy.

When asked if she ever doubted her abilities as a researcher Martha replied, “Not precisely, but rather there is the uncertainty generated by knowing that knowledge is infinite and that every day there are more and more ways to analyze a particular scientific question.” Professor Escobar is proud of a fulfilling career and pleased that women are now important contributors in the field as she states, “In recent years I have witnessed with satisfaction the large number of women who work in Neuroscience very successfully. More and more women are interested in science with very good performance, paving the way for the new generations. It is important that youth take ownership of what should really matter to all of us, at a time when humanity faces whether or not we have a future.”

## 3. Conclusion

With this brief compilation about four brilliant neuroscientists, we wanted to highlight the great contributions of Hispanic women in Neuroscience by recognizing the outstanding work they have done and continue to do in identifying anatomical, molecular and cellular mechanisms that underlie normal and pathological processes in the brain. Many Hispanic women in Neuroscience, even very successful investigators, encounter funding difficulties, prejudice, financial, geographical and political challenges. However, they continue to be inspiration for their students, mentees and colleagues.

## Data availability statement

The original contributions presented in this study are included in the article/supplementary material, further inquiries can be directed to the corresponding author.

## Ethics statement

Ethical review and approval was not required for the study on human participants in accordance with the local legislation and institutional requirements. The patients/participants provided their written informed consent to participate in this study. Written informed consent was obtained from the individual(s) for the publication of any potentially identifiable images or data included in this article.

## Author contributions

MS-A, AP, EM, and VM-C contributed to the conception and design and wrote sections of the manuscript. All authors contributed to manuscript revision, read, and approved the submitted version.

